# A literature review on the representativeness of randomized controlled trial samples and implications for the external validity of trial results

**DOI:** 10.1186/s13063-015-1023-4

**Published:** 2015-11-03

**Authors:** Tessa Kennedy-Martin, Sarah Curtis, Douglas Faries, Susan Robinson, Joseph Johnston

**Affiliations:** Kennedy-Martin Health Outcomes Ltd, 3rd Floor, Queensberry House, 106 Queens Road, Brighton, BN1 3XF UK; Eli Lilly and Company, Indianapolis, Indiana USA

**Keywords:** Randomized controlled trial, External validity, Generalizability, Real-world patients, Cardiology, Mental health, Oncology, Literature review

## Abstract

Randomized controlled trials (RCTs) are conducted under idealized and rigorously controlled conditions that may compromise their external validity. A literature review was conducted of published English language articles that reported the findings of studies assessing external validity by a comparison of the patient sample included in RCTs reporting on pharmaceutical interventions with patients from everyday clinical practice. The review focused on publications in the fields of cardiology, mental health, and oncology. A range of databases were interrogated (MEDLINE; EMBASE; Science Citation Index; Cochrane Methodology Register). Double-abstract review and data extraction were performed as per protocol specifications. Out of 5,456 de-duplicated abstracts, 52 studies met the inclusion criteria (cardiology, n = 20; mental health, n = 17; oncology, n = 15). Studies either performed an analysis of the baseline characteristics (demographic, socioeconomic, and clinical parameters) of RCT-enrolled patients compared with a real-world population, or assessed the proportion of real-world patients who would have been eligible for RCT inclusion following the application of RCT inclusion/exclusion criteria. Many of the included studies concluded that RCT samples are highly selected and have a lower risk profile than real-world populations, with the frequent exclusion of elderly patients and patients with co-morbidities. Calculation of ineligibility rates in individual studies showed that a high proportion of the general disease population was often excluded from trials. The majority of studies (n = 37 [71.2 %]) explicitly concluded that RCT samples were not broadly representative of real-world patients and that this may limit the external validity of the RCT. Authors made a number of recommendations to improve external validity. Findings from this review indicate that there is a need to improve the external validity of RCTs such that physicians treating patients in real-world settings have the appropriate evidence on which to base their clinical decisions. This goal could be achieved by trial design modification to include a more representative patient sample and by supplementing RCT evidence with data generated from observational studies. In general, a thoughtful approach to clinical evidence generation is required in which the trade-offs between internal and external validity are considered in a holistic and balanced manner.

**Electronic supplementary material:**

The online version of this article (doi:10.1186/s13063-015-1023-4) contains supplementary material, which is available to authorized users.

## Background

Appropriately designed and executed randomized controlled trials (RCTs) represent the current gold-standard primary study design for the determination of the efficacy and safety of medical interventions [1]. Evidence from RCTs is used by healthcare providers to guide their clinical decisions and by payers and policy makers to support their recommendations for the adoption of new therapies in clinical practice [2]. Explanatory RCTs are designed to determine the efficacy of an intervention under idealized and controlled circumstances and so are conducted under rigorous conditions, including strict adherence to structured protocols, the use of restrictive inclusion and exclusion criteria, and patient randomization, that maximize their internal validity (that is to ensure they minimize the possibility of bias regarding the effect of an intervention) [3, 4]. In order for the results of such trials to be clinically useful, they must also be relevant to a definable patient population in a specific healthcare setting, a concept that is termed external validity or generalizability (note, these terms are used interchangeably [3] in this review and describe the applicability of the study results outside of the trial environment) [5–7]. As it is challenging to simultaneously optimize internal and external validity, efficacy data from traditional explanatory RCTs are often complemented by evidence from pragmatic trials (including pragmatic RCTs) or observational studies that determine the performance of an intervention under conditions more closely resembling routine clinical practice, and include more heterogeneous patient populations and less stringent treatment and delivery protocols [4]. While some pragmatic trials have good internal validity and some observational studies may lack external validity, generally explanatory RCTs tend to maximize internal validity at the expense of external validity, while studies conducted in a setting more closely resembling real-world practice may do the opposite. As such, evidence from all these sources can be complementary in understanding the effect of an intervention and furthering clinical research [8].

In recent years, the need to better understand the external validity of RCT results has been identified across numerous therapeutic areas [9–13]. However, a comprehensive literature review of studies that have assessed the representativeness of RCT populations has not been undertaken in recent years (note, the term representativeness has been used throughout this review to describe the similarities between RCT samples and real-world populations). To examine this issue, we conducted a literature review of studies that have attempted to evaluate external validity in one of two ways: (i) by comparing the clinical characteristics of an RCT sample with those of everyday clinical practice patients, or (ii) by assessing what proportion of a real-world population would satisfy the criteria for RCT inclusion. In the context of the current review, real-world populations are defined as those patients encountered in routine clinical practice settings (for example, patients included in observational cohorts or patients identified from medical chart review, registries, or insurance databases). The primary objective of the review was to assess the extent to which RCT samples are representative of real-world populations (which may or may not affect the external validity of the trial findings). Other objectives were to identify key issues that may impact the external validity of trial findings (with reference to included studies) and also to outline recommendations from the identified studies for improving external validity. The present review was limited to RCTs in oncology, mental health, and cardiology as, when the review was undertaken, these were identified as the main therapeutic areas in which RCT and real-world populations had been compared. It should be noted that the focus of the current review was explanatory and not pragmatic RCTs.

## Review

### Methods

The methodological framework of this literature review was employed to examine the extent, range, and nature of research activity regarding the representativeness of RCT patient samples and the implications of this to the external validity of the findings. The review involved a five-stage process [14]: identification of the research question; identification of studies relevant to the research question; selection of studies to include in the review; charting of information and data within the included studies; collating, summarizing, and reporting results of the review. A search protocol was written that outlined the objectives, search methods, and the process for study selection and data extraction.

### Information sources, search approach, and strategy

Searches were run in MEDLINE and MEDLINE In-Process, EMBASE, Science Citation Index, and the Cochrane Methodology Register and were supplemented with reference checking. When combined with citation searching, these sources presented a reasonable basis for a targeted search of the published literature. The searches were run on 30 September 2013 and included published studies conducted from 2003 to 2013 in order to reflect contemporary clinical trial practice. A base-case search strategy was created in the Ovid MEDLINE interface, and once finalized, was adapted to meet the syntax of the other databases. See Additional file 1 for the full Ovid MEDLINE search.

Database searches were designed to identify primary research studies published in English providing an analysis of an adult (aged > 18 years) patient sample in an RCT (or number of RCTs or meta-analysis of RCTs) compared with an adult patient population treated outside of an RCT setting with the same condition. Studies could have quantitatively assessed how many patients in a real-world population would satisfy the eligibility requirements of an RCT, or compared the clinical characteristics of an RCT sample with a real-world population. Only those studies reporting on pharmaceutical interventions studied as part of an RCT (placebo-controlled or active comparator) were included. Case reports, methodology papers, and conference abstracts were not considered, nor were studies that undertook an analysis of patients who were recruited into an RCT compared with those that declined participation, or studies that involved a pediatric (aged < 18 years) population. This analysis was limited to studies in cardiology, mental health, and oncology, as the larger numbers of publications identified in these therapeutic areas allowed for a higher level synthesis of their findings.

### Study selection, data extraction, and reporting

Search results were assessed for relevance by two independent researchers by reviewing the title and abstract of all identified studies. Studies meeting or potentially meeting the review eligibility criteria were assessed in more detail using the full text. A third reviewer (TKM) resolved disagreements on study selection.

A data extraction table was developed and tested on a sample of studies before further refinement. Data were quality checked through double-data extraction by a second researcher on 10 % of the records included to ensure the format of data extraction tables was appropriate. All data included in the final manuscript were quality checked. The following data were extracted from each included publication: (i) generalizability objectives; (ii) patient populations and country of study; (iii) methods; (iv) description of RCT and real-world data sources; (v) listing of comparisons made and key results; (vi) overall conclusions; (vii) recommendations addressing identified issues and best practices.

Following a detailed review, a framework for the narrative analysis of the data was developed that included categorization of the identified studies by two methods. Method A involved a formal statistical comparison (for example, use of Wilcoxon rank sum test and chi-square test for continuous and categorical variables, respectively) of baseline characteristics between a real-world patient population and a patient sample enrolled in an RCT in the same specific disease area. Patients were compared for baseline characteristics such as demographics, clinical and disease data, and treatments and procedures. A range of different statistical methodologies were employed in the included studies, and it is outside of the scope of this review to detail them all; the reader is referred to the individual studies for more information. Method B involved a determination of the proportion of patients in a real-world population that would have been trial eligible or ineligible by review of individual patient medical records followed by the application of explicit eligibility criteria derived from specific RCTs or common criteria derived from a review of multiple RCTs in the same disease to individual patient data. The ineligibility rates as calculated in each individual Method B study were tabulated and the distribution by quartiles examined. A minority of studies employed a mixture of methods (A and B) and presentation of the findings from such studies was split by method (Tables 2 and 3).

In order to interpret the main findings of the literature review as they related to external validity, a qualitative synthesis of individual study results was undertaken. The discussion and conclusions of each publication were closely studied by one researcher, and the subjective author conclusions with respect to “external validity”, “generalizability”, or “representativeness” were tabulated. These were then grouped according to the precise wording used by individual authors and categorized as: “Different” if the authors explicitly commented that, in their opinion, there were meaningful differences between RCT samples and real-world populations that suggested they were not representative, that the data could not be extrapolated or were not applicable to real-world settings, and/or that external validity is impacted; “Not Explicit” if the authors did not explicitly comment on external validity or did not comment on external validity despite demonstration of differences in baseline characteristics; “Similar” if the authors commented that populations were similar and/or RCT results were generalizable to the overall disease population. A second researcher checked the grouping of each study by category; in the event of any disagreements, the findings of each paper were discussed until resolution was reached.

## Results

### Search results

**Fig. 1:**
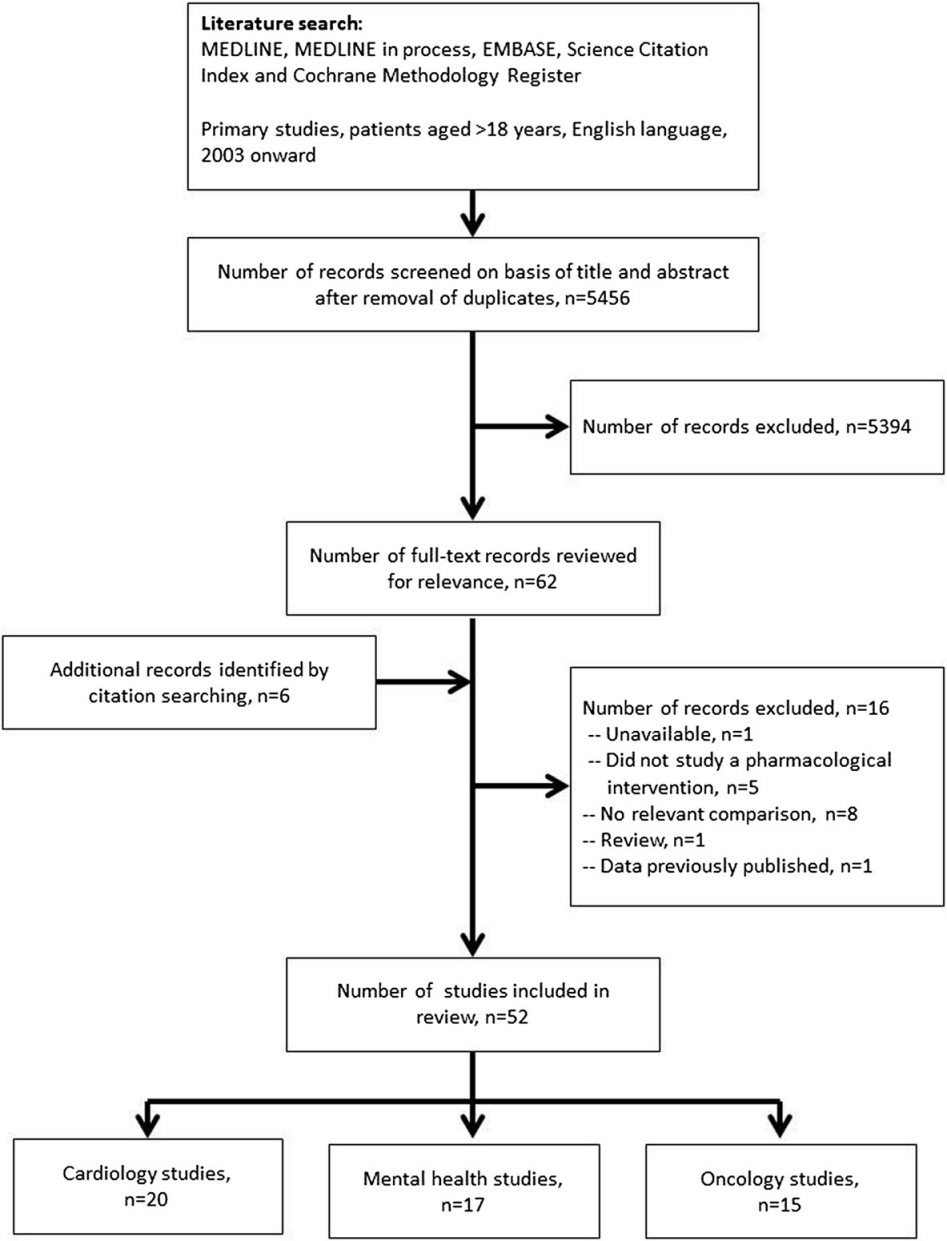
Study selection for a literature review assessing the external validity of randomized controlled trials

The study selection is shown in Fig. 1. The original search returned 5,456 studies of which 46 in the areas of cardiology, mental health, and oncology were identified as relevant after abstract review. An additional six studies were identified through citation searching.

### Study design

Of the 52 studies included, 18 (34.6 %) employed only Method A (comparison of baseline characteristics) while 27 (51.9 %) employed only Method B (determination of percentage ineligibility) (Table 1). An additional seven studies (13.5 %) used both Methods A and B. The highest number of studies was conducted in the USA (Table 1). The populations studied using Method A were compared for demographics, clinical characteristics, baseline treatments and procedures, and other variables (Table 1). Additional analyses were conducted in some Method B studies as detailed in Table 1. The sources and settings from which RCT samples and real-world patient populations were drawn are listed in Tables 2 and 3 (a more detailed summary of sources is provided in Additional file 2).

**Table 1:** Study design overview of included publications

	Number (%)			
	Cardiology	Mental health	Oncology	Total
Total number of studies	20 (38.5)	17 (32.7)	15 (28.8)	52 (100)
Geography				
USA	8^a^ (40.0)	10 (58.8)	5 (33.3)	23 (44.2)
The Netherlands	1 (5.0)	3 (17.6)	2 (13.3)	6 (11.5)
Germany	1 (5.0)	2 (11.8)	2 (13.3)	5 (9.6)
Canada	3 (15.0)	1 (5.9)	1 (6.7)	5 (9.6)
Other	7 (35.0)	1 (5.9)	5 (33.3)	13 (25.0)
Method^b^				
A only	9 (45.0)	2 (11.8)	7 (46.7)	18 (34.6)
B only	9 (45.0)	12 (70.6)	6 (40.0)	27 (51.9)
A and B	2 (10.0)	3 (17.6)	2 (13.3)	7 (13.5)
Comparisons made, Method A^c,d^				
Demographics	10 (90.9)	5 (100)	8 (88.9)	23 (92.0)
Clinical characteristics	8 (72.7)	5 (100)	7 (77.8)	20 (80.0)
Treatments and procedures	4 (36.4)	2 (40.0)	3 (33.3)	9 (36.0)
Other^e^	1 (9.1)	1 (20.0)	0 (0.0)	2 (8.0)
Additional analyses undertaken, Method B^d^				
Comparison of baseline characteristics, eligible vs ineligible patients	6 (54.5)	6 (40.0)	1 (12.5)	13 (38.2)
Common reasons for trial ineligibility	7 (63.6)	14 (93.3)	8 (100)	29 (85.3)

^a^Includes one study conducted in the USA and Canada. ^b^Method A, formal statistical comparison of baseline characteristics between a real-world patient population and patients enrolled in a randomized controlled trial (RCT) in the same disease area; Method B, determination of the proportion of real-world patients who would have been trial eligible or ineligible by review of individual patient medical records followed by application of RCT eligibility criteria. ^c^Each study made multiple comparisons. ^d^Percentages calculated based on total number of studies employing method (for example, Method A studies plus Method A/B studies). ^e^Other comparisons included physical activity relative to “others the same age” (n = 1 cardiology study) and personality traits (n = 1 mental health study)

**Table 2:** Key results and main author conclusions from Method A studies

Study	Real-world data source	Key differences (real-world versus RCT patients)	Main author conclusions^b^
Cardiology			
Badano et al., 2003 [15]	MC-PR	Older, more female, higher rates of concomitant diabetes, greater LVF clinical impairment	Different
Björklund et al., 2004 [17]	MC-PR	Older, more female and more CV risk factors	Different
Costantino et al., 2009^a^ [21]	SC-PR	Older, more female, lower NYHA class	Different
Dhruva et al., 2008 [22]	ID	Older, more female	Different
Ezekowitz et al., 2012 [24]	MC-PR	Older, more female, more co-morbidities/prior cancer	Different
Golomb et al., 2012 [27]	MC-PR	Increased self-rated physical activity with increasing age	Different
Hutchinson-Jaffe et al., 2010 [29]	MC-PR	Older, more female, more co-morbidities, less guideline-recommended treatment/procedures	Different
Melloni et al., 2010 [37]	MC-PR	More female	Different
Steinberg et al., 2007 [62]	MC-PR	Older, more co-morbidities/CVD history	NE
Uijen et al., 2007^a^ [44]	MC-PR	Older, more female, higher CVD risk	Different
Wagner et al., 2011 [65]	ID	Older, more chronic diseases	NE
Mental health			
Kushner et al., 2009 [57]	MC-PR	Greater depression severity (some scales), lower preference for novel experiences	NE
Rabinowitz et al., 2003^a^ [59]	MC-PR	No major differences	Similar
Riedel et al., 2005 [60]	SC-PR	Older, longer duration of illness, more internistic co-morbidities/hospitalizations	Similar
Surman et al., 2010^a^ [42]	SC-PR	More co-morbidities, anxiety/depression, alcohol/substance dependence	Different
Zarin et al., 2005^a^ [49]	MC-PR	Older, more female/Caucasian	Different
Oncology			
Baquet et al., 2009 [52]	MC-PR	Fewer females (non-sex-specific tumor RCTs), fewer males (sex-specific tumor RCTs)	NE
Elting et al., 2006 [23]	SC-PR	Older, more females/chronic co-morbidities, worse health/performance status	Different
Fraser et al., 2011^a^ [25]	MC-PR	Worse disease prognosis, more drug-related toxicity, lower drug dose intensity	Different
Jennens et al., 2006 [30]	MC-PR	Older	Different
Kalata et al., 2009 [31]	MC-PR	Older, more females, worse prognosis	Different
Mengis et al., 2003^a^ [38]	SC-PR	Older, worse performance status, more infections/AML-MDS subtypes	Different
van der Linden et al., 2014 [45]	MC-PR	Older, more females, poor prognostic factors	Different
Yennurajalingam et al., 2013 [48]	SC-PR	Older, more males, higher symptom intensity scores	Different
Yessaian et al., 2005 [66]	MC-PR	No major differences	Similar

Please see Additional files 2 and 3 for more detailed results

^a^Studies that employed Methods A and B; in these studies RCT and real-world populations were compared, the authors then used the eligibility criteria from the RCT of interest to determine how many patients would hypothetically have been eligible or ineligible for that trial. Results presented in this table are for Method A only (see Table 3 for Method B results). ^b^Different: authors explicitly comment, in their opinion, that there were meaningful differences between populations that suggested they were not representative, that the data could not be extrapolated or were not applicable to real-world settings, and/or that external validity is impacted; NE: authors do not explicitly comment on external validity or do not comment on external validity despite demonstration of differences in baseline characteristics; Similar: authors comment that populations are similar and/or that RCT results are generalizable to the overall disease population

*AML* acute myeloid leukemia, *CV* cardiovascular, *CVD* cardiovascular disease, *ID* insurance data; *LVF* left ventricular function, *MC*-*PR* patient records - multicenter (including multicenter registries), *MDS* myelodysplastic syndrome, *NYHA* New York Heart Association, *RCT* randomized controlled trial, *SC*-*PR* patient records - single center

**Table 3:** Key results and main author conclusions from Method B studies

Study	Real-world data source	% ineligibility^a^	Key differences (ineligible versus eligible patients)	Main author conclusions^b^
Cardiology				
Bahit et al., 2003 [16]	MC-PR	33.6	Older, more females/previous MI, lower ASA use, longer LOS	Different
Bosch et al., 2008 [19]	SC-PR	41.2	Older, higher risk profile	Different
Collet et al., 2003 [53]	SC-PR	34.0	Older, more females, higher risk score, fewer in-hospital procedures	NE
Costantino et al., 2009^c^ [21]	SC-PR	66.2	ND	Different
Fortin et al., 2006 [55]	MC-PR	1.4–65.5	ND	NE
Koeth et al., 2009 [34]	MC-PR	46.4	Older, more females, more diabetes/hypertension, less guideline-recommended treatment	Different
Krumholz et al., 2003 [56]	MC-PR	84.5 (NRMI)	ND	Similar
		90.6 (CCP)		
Lenzen et al., 2005 [35]	MC-PR	61.6	Older, more females, more co-morbid hypertension/ACS/renal impairment, less guideline-recommended treatment at baseline	Different
Masoudi et al., 2003 [36]	ID	67.0	ND	Different
Steg et al., 2007 [40]	MC-PR	33.6	Older, history of MI, diabetes, TIA, PAD, and CABG, less guideline-recommended treatment/procedures, high risk score	Different
Uijen et al., 2007^c^ [44]	MC-PR	53.0	ND	Different
Mental health				
Blanco et al., 2008 [18]	GP	75.8	ND	Different
Goedhard et al., 2010 [26]	SC-PR	69.8	Older, more Axis II personality disorders	Different
Hoertel et al., 2013 [28]	GP	58.2 (bipolar)	ND	Different
		55.8 (mania)		
Keitner et al., 2003 [32]	SC-PR	85.5	ND	Different
Khan et al., 2005 [33]	GP	98.2	ND	Different
Rabinowitz et al., 2003^c^ [59]	MC-PR	33.0	ND	Similar
Seemuller et al., 2010 [61]	MC-PR	69.0	Younger, trend to younger age at disease onset	Similar
Storosum et al., 2004 [41]	SC-PR	83.8^d^	ND	Different
Surman et al., 2010^c^ [42]	SC-PR	61.0	More lifetime co-morbidity, lower overall functioning/SES	Different
Talamo et al., 2008 [63]	SC-PR	77.6	Few differences	Similar
van der Lem et al., 2011 [64]	SC-PR	75.5–81.2^e^	ND	NE
Wisniewski et al., 2009 [47]	MC-PR	77.8	Older, less educated, more black/Hispanic, longer disease duration, history of suicide and substance abuse, more atypical features	Different
Zarin et al., 2005^c^ [49]	MC-PR	55.0 (bipolar) 38.0 (schizo-phrenia)	More co-morbidity, lower global functioning, greater use of antipsychotic medication	Different
Zetin and Hoepner, 2007 [50]	SC-PR	91.4	ND	Different
Zimmerman et al., 2004 [51]	SC-PR	65.8	ND	Different
Oncology				
Clarey et al., 2012 [20]	SC-PR	31.0–76.0	ND	Different
Filion et al., 2012 [54]	SC-PR	–^f^	ND	Similar
Fraser et al., 2011^c^ [25]	MC-PR	14.9	ND	Different
Mengis et al., 2003^c^ [38]	SC-PR	87.0	ND	Different
Mol et al., 2013 [58]	MC-PR	21.5	Worse performance status, higher alkaline phosphatase, less primary tumor resection	Similar
Somer et al., 2008 [39]	SC-PR	71.0	ND	Different
Terschüren et al., 2010 [43]	MC-PR	35.9 (HL)	ND	Different
		70.4 (hgNHL)		
Vardy et al., 2009 [46]	MC-PR	65.0–72.0	ND	Different

Please see Additional files 2 and 4 for more detailed results

^a^Percentage of patients not eligible for RCT inclusion following the application of eligibility criteria. ^b^Different: authors explicitly comment, in their opinion, that there were meaningful differences between populations that suggested they were not representative, that the data could not be extrapolated or were not applicable to real-world settings, and/or that external validity is impacted; NE: authors do not explicitly comment on external validity or do not comment on external validity despite demonstration of differences in baseline characteristics; Similar: authors comment that populations are similar and/or that RCT results are generalizable to the overall disease population. ^c^Studies that employed Methods A and B; in these studies RCT samples and real-world populations were compared, the authors then used the eligibility criteria from the RCT of interest to determine how many patients would hypothetically have been eligible or ineligible for that trial. Results presented in this table are for Method B only (see Table 2 for Method A results). ^d^Percentage of manic episodes not number of ineligible. ^e^75.5 % based on application of stringent criteria using the Mittman regression equation to calculate HAM-D; 81.2 % based on application of stringent criteria using the Hawley or Zimmerman regression equation to calculate HAM-D. ^f^Inclusion/exclusion criteria were categorized to identify criteria that might impede RCT recruitment; if any individual category was not met by > 10 % of patients with breast cancer from a retrospective cohort, then the criterion was considered a barrier to recruitment. *ACS* acute coronary syndrome, *ASA aspirin*, *CABG* coronary artery bypass graft, *CCP* cooperative cardiovascular project, *GP* general population data, *HL* Hodgkin’s lymphoma, *hgNHL* high-grade non-Hodgkin’s lymphoma, *ID* insurance data, *LOS *length of stay, *MC*-*PR* patient records - multicenter (including multicenter registries and observational studies), *MI* myocardial infarction, *ND* not determined, *NRMI* National Registry of Myocardial Infarction, *PAD* peripheral arterial disease, *SC*-*PR* patient records - single center, *SES* socioeconomic status, *TIA* transient ischemic attack

### Representativeness/external validity

In 37 (71.2 %) studies (12 [66.7 %] Method A; 19 [70.3 %] Method B; 6 [85.7 %] Method A/B), the individual study authors concluded that RCT samples were not representative of patients encountered in clinical practice and/or that population differences may have a relevant impact on the external validity of the RCT findings [15–51]. The remaining 15 studies [52–66] did not reach an explicit conclusion regarding external validity or concluded that populations were broadly similar, although we note that in some cases the authors still reported differences between RCT samples and real-world populations (Tables 2 and 3) [53, 57, 62, 64, 65].

### Cardiology

**Fig. 2:**
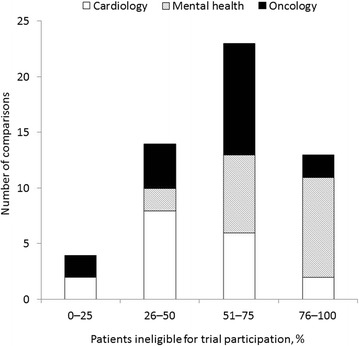
Proportion of real-world patients ineligible in randomized controlled trials (RCTs) after application of inclusion/exclusion criteria. Method B studies. Some individual studies reported multiple ineligibility rates derived from the application of selection criteria from a number of different RCTs to a single real-world population. Hence, in the 34 studies that employed Method B, 54 different ineligibility rates were calculated

Studies included in the review generally demonstrated that, compared with patients enrolled in major cardiology RCTs, patients encountered in everyday practice were more likely to have higher risk characteristics as they were older, more likely to be female and to have clinical impairment and co-morbid disease, were treated less frequently with guideline-recommended therapy, and received fewer in-hospital procedures (Table 2). When RCT inclusion/exclusion criteria were applied to real-world cardiology patients (Method B), those patients who would have been ineligible for RCT participation were more likely to be older and female, to have co-morbid disease, and to less frequently receive guideline-recommended therapy compared with patients who would have been eligible for the trial (Table 3). In 11 studies employing Method B, 18 different sets of eligibility criteria were applied to real-world populations and ineligibility rates reported; in eight cases (44.4 %) more than 50 % of patients were reported to be ineligible for trial inclusion (Fig. 2 and Table 3). The reasons for ineligibility varied considerably by study depending on the specific condition under assessment.

### Mental health

In general, the identified studies reported that real-world patients with mental health disorders tended to be more severely ill than patients enrolled in RCTs. They also appeared to have more co-morbidities and, in some cases, lower overall functioning and socioeconomic status (Table 2). Studies that assessed the characteristics of a real-world population after the application of specific RCT inclusion/exclusion (Method B) reported that patients who would have been RCT ineligible were older, had more co-morbidities and more severe disease, exhibited lower overall functioning, and had lower socioeconomic status than patients who would have been eligible for trial participation (Table 3). In the 15 studies employing Method B, 18 different sets of eligibility criteria were applied to real-world populations resulting in ineligibility rates in excess of 50 % in 16 (88.9 %) cases (Fig. 2 and Table 3). Common reasons for RCT exclusion across studies included current or history of substance abuse, suicide risk, presence of co-morbidities (such as other Axis I disorder, co-morbid anxiety, and other central nervous system [CNS] or neuromuscular disorder), insufficient symptom duration or low disease severity (in studies of major depressive disease), contraindicated medication, and significant medical condition (see Additional files 3 and 4).

### Oncology

Compared with RCT-enrolled patients, real-world patients with cancer were often older, and more likely to be female, have a poor performance status, and worse disease prognosis (Table 2) in the studies selected in this review. A single study compared the baseline characteristics between RCT ineligible versus eligible patients after the application of inclusion/exclusion criteria and found that ineligible patients with colorectal cancer had a worse performance status (Table 3) [58]. In the eight studies employing Method B, 18 different sets of eligibility criteria were applied to real-world populations, with ineligibility rates greater than 50 % being reported in 12 (66.7 %) cases (Fig. 2 and Table 3). Reasons for trial exclusion included poor performance status, previous history of cancer, co-morbidities, reduced life expectancy, CNS or brain metastases, and older age (see Additional files 3 and 4).

### Potential factors influencing the external validity of RCTs

In the majority of included studies, the authors made some attempt to identify factors influencing the external validity of RCTs. These could broadly be divided into explicit and implicit factors: explicit factors are the inclusion/exclusion criteria listed in the study protocol, while implicit factors include other issues that may affect patient participation in any given trial. The influence of implicit factors on external validity could only be hypothesized in the included studies and are outlined below.

### Explicit factors (restrictive inclusion/exclusion criteria)

Explicit factors were identified as a key driver for differences in RCT samples and real-world populations, as demonstrated by the often high rates of trial ineligibility (Fig. 2 and Table 3) determined in the included studies. By using restrictive inclusion/exclusion criteria, higher risk patients are effectively excluded from RCTs. For example, in cardiology studies, patients often appeared to be excluded on the basis of older age and presence of co-morbid disease. The authors of these studies suggested that cardiovascular disease may represent a more complicated syndrome in such patients [15] and that they are more likely to experience adverse events [16, 19]. As such, the results from these studies may not provide a complete picture of anticipated drug efficacy and safety in clinical practice. Female patients were also under-represented in the cardiology trials identified in this review [15, 17, 24, 29, 37]; one of the reasons for this may be due to cardiovascular disease affecting women later in life, meaning that upper age limit restrictions may disproportionately limit their inclusion in RCTs relative to men [37]. In mental health studies, high proportions of patients were excluded on the basis of substance abuse, which is a particular issue for the external validity of trials in bipolar patients where rates are high [41]. One study applied only the exclusion criteria that the authors considered strictly necessary with respect to safety and found that nearly 75 % of patients with depression were still ineligible for participation in efficacy RCTs [50]. Patient samples in oncology trials were often found to have better disease prognosis and better performance status compared with real-world patients with cancer [23, 25, 31, 38, 45]. Inadequate performance status (for example, Eastern Cooperative Oncology Group performance status ≥ 2) was one of the most common reasons for trial exclusion in several studies [20, 39, 46, 58].

### Implicit factors

Implicit factors that may have affected the external validity of RCTs were also identified in some of the studies reviewed. Two cardiology studies noted that issues with informed consent, whereby the most severely ill patients are less likely to give informed consent or it is harder to gain informed consent, may lead to the selection of lower risk patients for trial participation [16, 17]. In addition, one study indicated that psychiatric patients with more severe aggression were also less likely to consent to enter an RCT [26]. The type of RCT setting and/or recruitment method were also discussed as potential barriers to trial participation [26, 33, 49]; for example, one study that evaluated how many patients with schizophrenia would be eligible for antipsychotic clinical trials suggested that there could be discrepancies between subjects who were recruited through advertisement and those recruited in a clinical setting [33]. In oncology patients (and their physicians), one of the biggest barriers to trial participation was noted to be fear of randomization to the placebo arm [43]. A number of other patient-related factors were also identified, including logistical issues related to study participation, beliefs and attitudes regarding the safety of trial medications, cultural factors, level of satisfaction with current treatment, and willingness to participate [39, 43, 48, 49]. Finally, one study demonstrated that patients who participate in trials may have different personality traits than those who do not; patients with depression who were enrolled in an antidepressant medication RCT were found to score more highly on a personality scale that assessed preferences for novel experiences compared with non-participants [57].

### Study recommendations for the improvement of external validity

Many of the studies included in the present review made recommendations to improve the external validity of RCTs. These recommendations are outlined in Table 4 and include modifying RCT design to improve external validity directly, and generating complementary evidence from alternative study types to address the limited external validity of the RCT post hoc.

**Table 4:** Recommendations for managing external validity issues made by included studies

Patient populations
Broadening of RCT inclusion and exclusion criteria [19, 20, 29, 31–33, 36, 38, 40, 42, 44, 47, 49]
Selection of patients from more appropriate settings/populations to achieve a more representative sample (for example, prospective use of registry data; a priori estimation of patient eligibility by application of trial exclusion criteria to the target population) [15, 17, 18, 31, 44, 54]
Conduct of RCTs in specific patient subgroups [20, 28, 30, 31, 46]
Standardization of inclusion/exclusion criteria and diagnostic and screening assessments across RCTs in a given medical condition [51]
Intervention
Broader range of RCT treatments (that is, different and realistic dosing regimens, use of concurrent therapy, and appropriate duration of treatment); comparison of new treatments with treatments as usual rather than to a prescribed dose of a particular medicine [49]
Reporting
Improved reporting of populations and results (that is, greater transparency in the reporting of how exclusion criteria are operationalized and how this influences eligibility, and of the rate and major characteristics of excluded patients) [28, 38, 51]
Collection, reporting, and comparison of data from patients within and outside of the trial [24, 28, 63]
Analysis
Development of statistical analysis plans and power calculation adjustment to ensure adequate powering for subgroup analyses [20, 37]
Generation of supportive data
Conduct of observational studies after the demonstration of treatment efficacy at the RCT level [15, 23, 36]
Development of large patient registries in specific disease areas [19]
Adoption of pragmatic studies [48, 49]
Clinical practice recommendations
Prospective auditing of drug efficacy and safety in everyday practice settings and comparison of these data with RCT results [25]
Provision of more detailed product information to include the criteria by which patients were selected in pivotal RCTs [20]

*RCT* randomized controlled trial

## Discussion

The present analysis utilized a robust literature review methodology to identify studies that compared the clinical characteristics of an RCT sample and patients from a real-world source (Method A) or assessed the proportion of a real-world population that would satisfy criteria for RCT inclusion (Method B). Publications identified by this methodology indicated that RCT samples in cardiology, mental health, and oncology studies that assessed pharmaceutical interventions in adult patients were often not broadly representative of patients treated in everyday clinical practice and that caution should be exercised when extrapolating data from trials to patients treated in usual care settings. Note that, with the exception of a single study [40], none of the RCTs described in the included studies were documented as being of a pragmatic design. In this Method B study, the RCTs in acute coronary syndrome from which eligibility criteria were extracted were described as having pragmatic enrollment strategies; however, the analysis still suggested that there were important differences in risk profile between RCT eligible and ineligible patients [40]. Differences in demographics, clinical characteristics, and treatments and procedures were reported between RCT and real-world patients by studies that employed Method A in their analyses [15, 17, 21–25, 27, 29–31, 37, 38, 42, 44, 45, 48, 49]. Similarly, when specific RCT inclusion/exclusion criteria were applied to real-world populations (Method B), important differences with respect to demographics and clinical and treatment parameters were identified between patients who would have been RCT ineligible compared with those who would have been eligible for the trial [16, 18–21, 25, 26, 28, 32–36, 38–44, 46, 47, 49–51]. Furthermore, it was observed that large proportions of the general disease population were often excluded from trial participation. We note that some differences in generalizability were observed between the different therapeutic areas studied in the present review.

In only a minority of studies did the authors conclude that RCT samples were broadly representative of real-world populations and that external validity was not impacted, or failed to reach an explicit conclusion regarding external validity despite demonstrating some differences in baseline characteristics between groups [52–66]. These findings are largely consistent with a previously published systematic sampling review that assessed the nature and extent of exclusion criteria among RCTs published between 1994 and 2006 in selected medical journals with impact factors > 2.5 [2]. While involving the review of older studies and use of more restrictive search criteria than the present review, this earlier study also demonstrated that RCTs often exclude large proportions of the general disease population and specific patient groups from trial participation. In agreement with the present review, it was reported that the elderly, women, and patients with co-morbidities were frequently ineligible for trial inclusion [2]. However, note that RCT findings may still be externally valid even in circumstances where the patient sample is not broadly representative of the real-world population. For example, one study included in the present review concluded that patients with unstable angina or non-ST-segment elevation myocardial infarction who would have been excluded from enoxaparin RCTs could be safety treated in clinical practice [53].

That the external validity of RCT results is often limited is widely acknowledged by clinicians as a problem when it comes to extrapolating data to the patients seen in everyday practice [3, 7]. Indeed, it is an often-cited reason for the frequent underuse of guideline-recommended therapies [67]. Where there is no evidence of efficacy in specific patient groups, clinicians may well be right in withholding treatment so as to prevent unanticipated harm [35]. This situation could, however, mean that patients at highest baseline risk who might be expected to receive the most benefit from a particular therapy are undertreated. This so-called “treatment-risk paradox” has been well described, particularly in cardiology [6].

In the studies included in the present review, the use of restrictive inclusion/exclusion criteria in RCTs was identified as being one of the key factors that limited the external validity of trial findings. Authors reported that frequently excluded patients were the elderly, females, or those with co-morbidities in cardiology studies [15–17, 19, 24, 29, 34, 35, 40, 44, 53, 55], patients with evidence of substance abuse or co-morbid psychological disorders in mental health studies [18, 28, 32, 33, 41, 42, 47, 49, 50, 61, 64], and patients with poor disease prognosis in oncology studies [20, 25, 31, 38, 39, 45, 46]. These RCT populations were, therefore, often highly selected and represented a patient sample at much lower risk of adverse events and complications compared with patients in clinical practice. The use of stringent selection criteria in RCTs ensures a homogeneous patient sample, optimizes internal validity of the study by reducing variance and removing potential confounding, so increasing the likelihood of finding a true association between treatment exposure and outcomes (that is, it makes it easier to distinguish the “signal” [treatment effect] from the “noise” [bias and chance]) [68, 69]. While the use of highly selected populations does not necessarily imply that a given treatment under study would fail to have equivalent efficacy and safety in under-represented patient groups, it does create uncertainty that can only be dispelled through the generation of additional evidence. However, it is pertinent to also consider how inclusion of high-risk patients may affect the outcomes of traditional trials. Patients with more co-morbidities or co-interventions may be more likely to prematurely discontinue study participation, which could lead to high attrition rates and a negative impact on trial validity and outcomes.

The studies reviewed herein made several recommendations to either improve the external validity of RCTs or compensate for limitations thereof. These included adaptation of trial designs to include a more heterogeneous patient sample that better represents different subgroups such as the elderly or patients with co-morbidities [19, 20, 28–33, 46]. Some studies suggested that adoption of pragmatic trial designs may be a way forward [48, 49]. Traditional RCTs are often described as “explanatory” trials since they aim to evaluate treatment efficacy under idealized conditions, and to explore “if and how an intervention works”. In contrast, pragmatic trials evaluate the effects of an intervention under usual conditions and their designs seek to determine “if an intervention actually works in real-life” [70]. In recent years, the Pragmatic–Explanatory Continuum Indicator Summary (PRECIS) tool has been developed, and has now been updated with the PRECIS-2 version to allow trialists to design studies that better support the needs of the intended users of the results. PRECIS-2 consists of nine domains (including “participant eligibility criteria”) in which design decisions are made to determine the extent to which the trial is pragmatic or explanatory, and to help ensure that the design achieves the primary purpose of the trial [71]. In addition to its application as an aid to trial design, PRECIS-2 has the potential for use in the assessment of completed trials for methodological quality and the likelihood of outcome bias in much the same way as the current Grading of Research, Assessment, Development and Evaluation (GRADE) system is used to assist guideline developers.

There is growing interest in different analytical methods that utilize data from multiple studies to extend and complement the evidence provided by a single clinical trial. Meta-analysis [72, 73] can be used to combine evidence from multiple clinical trials to provide a more valid estimate of treatment effect, assuming the studies being combined are similar enough to permit synthesis. Cross-design synthesis is a type of meta-analysis in which evidence from studies with complementary designs are combined in an effort to leverage complementary strengths (such as internal validity of RCTs and external validity of observational studies) and minimize the weaknesses of each [74]. Another approach that leverages real-world data to extend findings from a traditional trial involves development of propensity scores that predict, for each trial subject, membership in a corresponding real-world population [75, 76]. Subjects over-represented in the clinical trial relative to the target real-world population receive lower weights while those under-represented receive higher weights. The resulting weights can be used to understand differences between the trial and target real-world populations, and to “project” the RCT efficacy to the target population, in effect providing an estimate of the efficacy that would be observed were the trial to be conducted in a more representative everyday practice population [75, 76]. Finally, simple descriptive analysis of real-world data can also be employed in the trial planning stages to better understand the impact of specific design decisions (for example, potential exclusion criteria) on the anticipated generalizability of the trial results and so improve design. Adaptation of statistical analysis plans was recommended by two of the studies reviewed here as a method to facilitate analysis of important patient subgroups [20, 37].

Several of the reviewed studies highlighted incomplete reporting as a potential issue for the external validity of RCTs [24, 28, 38, 51, 63]. Improvements in trial reporting to provide a more detailed description of RCT samples would enable clinicians to better assess the external validity of RCTs and so more accurately extrapolate trial findings to their own patients. Following reporting guidelines such as CONSORT, which is a requirement for publication in many peer-reviewed journals [1], may go some way to address issues of inconsistent reporting and may provide greater transparency with respect to trial eligibility.

Trials should follow the need for evidence but be part of a broader strategy for evidence generation. As such, complementary data obtained from other appropriately designed alternatives conducted in Phase IV of the development lifecycle are required to address limitations in the external validity of RCTs post hoc. As recommended by some of the studies included in this review [15, 23, 36], the use of non-randomized observational studies that utilize large healthcare databases can support RCT findings by determining treatment effectiveness in routine clinical practice [6, 77]. Such studies include a wide range of different designs including prospective and retrospective cohort studies, case–control studies, and cross-sectional studies in which any intervention studied is determined by clinical practice and not a rigid protocol [78]. Taken together, RCT and observational study data should provide a complementary body of evidence that optimizes both internal and external validity.

The findings presented in this review must be viewed within the limitations of the methodology employed. Firstly, the search strategy did not define the outcomes to be reported a priori and was influenced by the evidence base identified. Secondly, there are no acknowledged methods for the assessment of the quality of data for this type of analysis. Thirdly, the present review was limited to just three therapeutic areas (cardiology, mental health, and oncology), and while a large proportion of the relevant literature was focused in these areas, it is possible that findings may be different in other specialties. In addition, to manage the scope of the review, we restricted our eligibility criteria to studies that included adults and assessed pharmaceutical interventions only, and we cannot completely rule out the possibility that findings might be different in pediatric populations or other healthcare interventions. Finally, the conclusions regarding external validity, as reported in individual studies, were subjective, which limited our ability to more accurately synthesize and summarize the findings. The review strategy was, however, relevant to the objective of the present analysis, as it utilized a robust and transparent approach in order to identify key concepts and the main sources of information available on the representativeness of RCT patient samples and the external validity of RCT findings. The framework for categorizing the methods used in individual studies and for interpreting individual study conclusions was consistent and clearly detailed, adding to the methodological rigor of the review.

## Conclusions

In the majority of studies included in this literature review it was concluded that patient samples in cardiology, mental health, and oncology RCTs are not broadly representative of patients encountered in everyday practice. These findings suggest that, while explanatory RCTs still represent the gold-standard primary study design for the generation of clinical efficacy evidence, there is a need to improve their external validity and/or supplement their results with data from a range of research approaches such that physicians treating patients in real-world settings have the appropriate evidence on which to base their clinical decisions and to provide greater insight regarding clinical effectiveness in everyday practice. This goal could be achieved in two ways: (i) modification of trial designs to include a patient sample more representative of the individuals expected to receive an intervention in real life, while recognizing the potential compromise of internal validity caused by increasing heterogeneity as discussed above [68, 69]; and (ii) supplementing RCT evidence with data generated from a continuum of appropriately designed supportive studies with alternative methodologies. In general, a thoughtful approach to RCT design is required in which the trade-offs between internal and external validity are considered in a holistic and balanced manner so that the results can better meet the diverse needs of regulators, prescribers, payers, and patients.

## Additional files

Additional file 1:
**Full Ovid MEDLINE search strategy for literature searches.** (PDF 280 kb) Additional file 2:
**Summary of real-world and RCT data sources employed in included studies.** Detailed description of data sources (real-world and RCT) used in studies included in review. (PDF 207 kb) Additional file 3:
**Key results and author conclusions from studies that compared baseline characteristics between a real-world patient population and a patient sample enrolled in an RCT (Method A).** Detailed description of results and subjective author conclusions from studies included in the review that employed Method A. (PDF 207 kb) Additional file 4:
**Key results and main author conclusions from studies assessing rates of ineligibility for RCT participation in a real-world patient population (Method B).** Detailed description of results and subjective author conclusions from studies included in the review that employed Method B. (PDF 227 kb)

## Abbreviations

CNS: central nervous system
PRECIS: Pragmatic–Explanatory Continuum Indicator Summary
RCT: randomized controlled trial
