# Supplementary material for: A literature review on the representativeness of randomized controlled trial samples and implications for the external validity of trial results
Source: Trials. 2015 Nov 3;16:495. doi: 10.1186/s13063-015-1023-4 (PMC4632358; doi:10.1186/s13063-015-1023-4)
Supplement: Additional file 1: — Full Ovid MEDLINE search strategy for literature searches. (PDF 280 kb) [file 13063_2015_1023_MOESM1_ESM.pdf]

Additional file 1

Full OVID MEDLINE search

Ovid MEDLINE(R) In-Process & Other Non-Indexed Citations and Ovid MEDLINE(R) 1946 to Present

#	Searches	Results	Search Type
1	exp Clinical Trials as Topic/	294121	Advanced
2	(randomised or randomized or rct or rcts or clinical trial$).ti,ab.	551208	Advanced
3	or/1-2	726830	Advanced
4	*"Reproducibility of Results"/	1543	Advanced
5	(external valid$ or externally valid$).ti,ab.	4655	Advanced
6	(generalisab$ or generalizab$).ti,ab.	9630	Advanced
7	(real world or real life or real patient$ or real practice$ or real clinical$ or real population$).ti.	3327	Advanced
8	(actual world or actual life or actual patient$ or actual practice$ or actual clinical$ or actual population$).ti.	167	Advanced
9	(transferable or transferability).ti,ab.	6743	Advanced
10	(unrepresentative or un-representative or "not representative" or "does not represent" or "do not represent").ti,ab.	5398	Advanced
11	(unreflective or un-reflective or "not reflective" or "does not reflect" or "do not reflect").ti,ab.	4864	Advanced
12	directness.ti,ab.	173	Advanced
13	(applicab$ or generaliz$ or generalis$).ti.	27827	Advanced
14	(reproducib$ or relevan$ or reality or realities or everyday or represent$).ti.	80008	Advanced
15	or/4-14	142233	Advanced

16	3 and 15	7915	Advanced
17	(aa or ad or ag or ai or de or dt or pd or pk or po or to or tu).fs.	5765649	Advanced
18	exp Drug Therapy/	1111794	Advanced
19	exp Pharmaceutical Preparations/	640602	Advanced
20	exp Drug Interactions/	145633	Advanced
21	(drug or drugs or pharmaceutical$1 or pharmacotherap$ or pharmaco-therap$ or chemotherap$ or chemo-therap$ or pharmacolog$ or medicin$ or medicat$ or agent$1 or dose$1 or dosage$1 or dosing).ti,ab.	3221521	Advanced
22	or/17-21	7275108	Advanced
23	16 and 22	4439	Advanced
24	(news or editorial or letter or comment or case reports).pt.	2948552	Advanced
25	case report.ti.	154821	Advanced
26	animals/ not humans/	3939518	Advanced
27	23 not (24 or 25 or 26)	4094	Advanced
28	limit 27 to (english language and yr="2003 -Current")	2792	Advanced
