# Supplementary material for: A literature review on the representativeness of randomized controlled trial samples and implications for the external validity of trial results
Source: Trials. 2015 Nov 3;16:495. doi: 10.1186/s13063-015-1023-4 (PMC4632358; doi:10.1186/s13063-015-1023-4)
Supplement: Additional file 2: — Summary of real-world and RCT data sources employed in included studies. Detailed description of data sources (real-world and RCT) used in studies included in review. (PDF 207 kb) [file 13063_2015_1023_MOESM2_ESM.pdf]

Additional file 2. Summary of real-world and RCT data sources employed in included studies

Study	Real-world sources	RCT sources
**Cardiology**		
Badano et al, 2003 [15]	Inpatients or outpatients with HF included in eight published epidemiological studies	Patients enrolled in 27 published RCTs cited in HF clinical guidelines
Bahit et al, 2003 [16]	TIMI9 registry patients with STEMI not eligible for TIMI9B trial inclusion	TIMI9B-enrolled patients with STEMI; eligibility criteria from TIMI9B
Björklund et al, 2004 [17]	Patients with MI included in a Swedish register of cardiac intensive care not enrolled in an RCT	Patients with STEMI enrolled in a double-blind RCT
Bosch et al, 2008 [19]	Patients with NSTEACS included in a prospective observational study	Eligibility criteria from RCTs on the efficacy of intravenous antithrombotic therapy in patients with NSTEACS
Collet et al, 2003 [53]	Patients with UA/NSTEMI included in an ischemic coronary	Seven exclusion criteria from two thrombolysis RCTs

Study	Real-world sources	RCT sources
	syndrome registry	
Costantino et al, 2009 [21]	Patients referred to an HF outpatient clinic	Patients included in 16 HF RCTs identified by literature search; eligibility criteria from published HF RCTs
Dhruva et al, 2008 [22]	Medicare beneficiaries	Patients included in studies forming the evidence base for cardiovascular technology assessments
Ezekowitz et al, 2012 [24]	Patients included in an acute HF registry (multicenter, retrospective, observational cohort) not enrolled in an RCT	Patients in a double-blind RCT in decompensated HF
Fortin et al, 2006 [55]	Patients included in a representative database of primary care family doctors	Eligibility criteria used in five RCTs in patients with hypertension listed in management guidelines
Golomb et al, 2012 [27]	Subjects included in a population-based observational study on the prevalence of arterial and venous disease	Subjects enrolled in a double-blind RCT on use of statins in a primary-prevention sample

Study	Real-world sources	RCT sources
Hutchinson-Jaffe et al, 2010 [29]	Patients hospitalized for ACS included in four registries (prospective, multicenter, observational studies) not participating in an RCT	RCT-enrolled patients included in the same four registries
Koeth et al, 2009 [34]	Patients with STEMI included in a prospective, multicenter, observational data pool	Eligibility criteria from RCTs in patients with STEMI
Krumholz et al, 2003 [56]	Patients with MI included in two registries who met eligibility criteria for an RCT on thrombolysis treatments	Patients enrolled in the thrombolysis RCT; eligibility criteria from the thrombolysis RCT
Lenzen et al, 2005 [35]	Patients with HF enrolled in the Euro Heart Survey	Eligibility criteria from three large RCTs studying effects of ACEIs, β-blockers, and aldosterone antagonists on mortality
Masoudi et al, 2003 [36]	National cohort of patients surviving hospitalization for HF	Eligibility criteria from three RCTs in HF

Study	Real-world sources	RCT sources
Melloni et al, 2010 [37]	General population with defined cardiovascular diseases (AHA Heart Disease and Stroke Statistics, 2008)	Patients enrolled in 156 RCTs cited by the AHA 2007 Women's Prevention Guidelines
Steg et al, 2007 [40]	Patients hospitalized with STEMI included in a registry	RCT-enrolled patients included in the registry; eligibility criteria from three RCTs in coronary reperfusion therapy
Steinberg et al, 2007 [62]	Patients with STEMI included in a registry	RCT-enrolled patients included in the same registry
Uijen et al, 2007 [44]	Patients with hypertension included in an Academic Network practice population	Patients in RCTs included in Dutch, British, and European hypertension guidelines; eligibility criteria from these RCTs
Wagner et al, 2011 [65]	Patients who underwent CABG at medical centers actively participating in an RCT who were eligible but not enrolled in the study	Patients enrolled in a multicenter CABG surgery RCT
**Mental health**		

Study	Real-world sources	RCT sources
Blanco et al, 2008 [18]	Patients with MDD included in a large nationally representative sample of the US adult population	Common exclusion criteria from efficacy RCTs in MDD
Goedhard et al, 2010 [26]	Inpatients with aggression in three long-stay wards at a mental health care institute	Exclusion criteria from 31 RCTs studying management of aggression as identified by literature search
Hoertel et al, 2013 [28]	Patients with DSM-IV bipolar depression or mania included in a large nationally representative sample of the US adult population	Seven most common exclusion criteria used in RCTs of bipolar depression and acute mania identified by literature review
Keitner et al, 2003 [32]	Patients who inquired about participating in one of two antidepressant efficacy trials	Eligibility criteria from two RCTs in MDD
Khan et al, 2005 [33]	General US population from the US Census Bureau, 1998	Eligibility criteria from an open-label, multi-dose study in schizophrenia

Study	Real-world sources	RCT sources
Kushner et al, 2009 [57]	Patients with MDD attending a depression clinic	Patients with MDD enrolled in an antidepressant efficacy RCT
Rabinowitz et al, 2003 [59]	Patients enrolled in an epidemiological study of patients with first-episode psychosis	Patients with first episode psychosis included in a large multinational RCT; exclusion criteria used in the same trial
Riedel et al, 2005 [60]	Patients with schizophrenia matched with RCT patients for DSM-IV diagnosis, gender, and age	Inpatients with a diagnosis of DSM-IV schizophrenia who had participated in Phase III RCTs on neuroleptics
Seemuller et al, 2010 [61]	Hospitalized patients with MDD (DSM-IV)	Common exclusion criteria from 31 RCTs identified by a systematic literature review
Storosum et al, 2004 [41]	Hospitalized patients with manic or mixed episode of bipolar disorder	Twelve inclusion and exclusion criteria from nine RCTs included in an acute manic episode registration dossier
Surman et al, 2010 [42]	A community sample of adult patients with ADHD included	Patients with ADHD participating in a double-blind RCT;

Study	Real-world sources	RCT sources
	in a naturalistic study	eligibility criteria from the same trial
Talamo et al, 2008 [63]	Inpatients prescribed antipsychotic, antimanic, or mood-stabilizing medicines	Eligibility criteria commonly reported in 21 published RCTs identified by literature search
van der Lem et al, 2011 [64]	Treatment-seeking outpatients with MDD from a regional healthcare setting	Common exclusion criteria used in antidepressant efficacy trials identified by literature review
Wisniewski et al, 2009 [47]	Outpatients enrolled in a prospective study in nonpsychotic MDD	Eligibility criteria used in placebo-controlled drug registration RCTs
Zarin et al, 2005 [49]	Patients with bipolar disorder or schizophrenia from a Practice Research Network study	Patients from two key published RCTs in bipolar I disorder or schizophrenia cited in practice guidelines; exclusion criteria used in the trials
Zetin and Hoepner, 2007 [50]	Patients with MDD or bipolar disorder from a single	Eleven RCT exclusion criteria derived from 31

Study	Real-world sources	RCT sources
	outpatient fee-for-service private practice	antidepressant efficacy studies
Zimmerman et al, 2004 [51]	Outpatients with MDD from a community-based practice	Exclusion criteria from 39 published antidepressant efficacy trials
**Oncology**		
Baquet et al, 2009 [52]	Patients included in the Maryland Cancer Registry	Patients included in the Maryland Cancer Registry who were accrued into NCI-sponsored cancer treatment clinical trials in 1999–2002
Clarey et al, 2012 [20]	Patients with advanced NSCLC (stages IIIB and IV) presenting at a large teaching hospital	Available eligibility criteria from nine Phase III NSCLC targeted therapy trials
Elting et al, 2006 [23]	Patients with newly-diagnosed cancer presenting at a University Hospital cancer center	Patients presenting at a University Hospital cancer center who participated in cancer treatment RCTs

Study	Real-world sources	RCT sources
Filion et al, 2012 [54]	Patients with invasive, nonmetastatic breast cancer included in the database at a specialized breast cancer center	Exclusion criteria from nine breast cancer Phase II or III RCTs in the adjuvant and neoadjuvant settings
Fraser et al, 2011 [25]	Patients with breast cancer commencing adjuvant FE100C-D chemotherapy at a regional cancer center and a general hospital	Patients with breast cancer enrolled in an adjuvant chemotherapy RCT; eligibility criteria from the same trial
Jennens et al, 2006 [30]	Patients included in a malignant neoplasm database	Patients with NSCLC or colorectal cancer enrolled in large RCTs
Kalata et al, 2009 [31]	Patients with stage II or III rectal cancer included in the Rostock Cancer Registry	Patients with locally advanced rectal cancer enrolled in an RCT
Mengis et al, 2003 [38]	Patients with AML diagnosed at a University Hospital	Patients with AML treated in five Phase III AML trials;

Study	Real-world sources	RCT sources
	treated outside of a clinical trial protocol	eligibility criteria from the trials
Mol et al, 2013 [58]	Patients with stage IV colorectal cancer receiving chemotherapy included in the Netherlands Cancer Registry	Patients included in a large Dutch Phase III trial for the treatment of advanced colorectal cancer; eligibility criteria from the trial
Somer et al, 2008 [39]	Treatment-naïve patients with advanced stage IV and 'wet' stage III NSCLC at the Fox Chase Cancer Center	Eligibility criteria for a unique NSCLC Phase III drug efficacy RCT
Terschüren et al, 2010 [43]	Incident cases of HL and hgNHL in a large population-based epidemiological survey	Patients included in six German multicenter HL RCTs or one multicenter hgNHL RCT; eligibility criteria from the trials
van der Linden et al, 2013 [45]	Patients with LA SCCHN included in a retrospective observational study	Patients with LA SCCHN included in a Phase III RCT

Study	Real-world sources	RCT sources
Vardy et al, 2009 [46]	Patients with stage IIIB (pleural effusion) or stage IV NSCLC registered in a cancer center database	Eligibility criteria for three Phase III chemotherapy RCTs
Yennurajalingam et al, 2013 [48]	Outpatients with advanced locally recurrent or metastatic cancer and fatigue at a palliative care clinic	Patients enrolled in five cancer-related fatigue clinical trials
Yessaian et al, 2005 [66]	Patients with cervical cancer from the NCI SEER database	Patients with primary cervical cancer included in eight Phase III RCTs conducted by large US cooperative groups

ACEIs: angiotensin-converting enzyme inhibitors; ADHD: attention deficit hyperactivity disorder; AHA: American Heart Association; AML: acute myeloid leukemia; CABG: coronary artery bypass graft; DSM-IV: Diagnostic and Statistical Manual of Mental Disorders; FE100C-D: six cycles of 5-flurouracil, epirubicin, and cyclophosphamide followed by three cycles of docetaxel; HF: heart failure; HL: Hodgkin's lymphoma; hgNHL: high-grade nonHodgkin's lymphoma; LA SCCHN: locally-advanced squamous cell carcinoma of the head and neck; MDD: major depressive disorder; MI: myocardial infarction; NSCLC: nonsmall cell lung cancer; NCI: National Cancer Institute; NSTEACS: Non-ST-segment elevation acute coronary syndrome; NSTEMI: Non-ST-segment elevation myocardial infarction; RCT:

randomized controlled trial; SEER: Surveillance, Epidemiology, and End Results; STEMI: ST-elevation myocardial infarction; TIMI: Thrombolysis in Myocardial Infarction; UA: unstable angina.
