# Supplementary material for: A literature review on the representativeness of randomized controlled trial samples and implications for the external validity of trial results
Source: Trials. 2015 Nov 3;16:495. doi: 10.1186/s13063-015-1023-4 (PMC4632358; doi:10.1186/s13063-015-1023-4)
Supplement: Additional file 3: — Key results and author conclusions from studies that compared baseline characteristics between a real-world patient population and a patient sample enrolled in an RCT (Method A). Detailed description of results and subjective author conclusions from studies included in the review that employed Method A. (PDF 207 kb) [file 13063_2015_1023_MOESM3_ESM.pdf]

Additional File 3. Key results and author conclusions from studies that compared baseline characteristics between a real-world patient population and a patient sample enrolled in an RCT (Method A)

Study	Key results (real-world patients vs RCT-enrolled patients)	Main author conclusions
**Cardiology**		
Badano et al, 2003 [15]	Real-world patients with HF were older and more likely to be female, and had higher rates of concomitant diabetes and more severe clinical impairment with respect to left ventricular function	Most of the RCTs on which guidelines for clinical practice have been based recruit patients that are not representative of patients with chronic HF treated in real-world practice
Björklund et al, 2004 [17]	Real-world patients with STEMI were older and more likely to be female and have more risk factors for cardiovascular disease (including previous MI, HF, and	There is a need for more representative enrollment in clinical trials of acute MI

Study	Key results (real-world patients vs RCT-enrolled patients)	Main author conclusions
	diabetes)	
Costantino et al, 2009[a] [21]	Outpatients with HF were older and more likely to be female and have a lower NYHA class	Differences exist between RCT patients and outpatients with HF suggesting that patient selection plays a crucial role in the applicability of RCT results
Dhruva et al, 2008 [22]	Medicare beneficiaries were older and more likely to be female	Data in cardiovascular technology assessments are derived from populations that differ significantly from a real-world Medicaid beneficiary population
Ezekowitz et al, 2012 [24]	Registry patients with HF were more likely to be older, female, and have co-morbidities, prior cancer, or an implantable cardiac device; SBP was higher,	RCTs select inpatients by inclusion criteria whereas registry data have broader generalizability, but may lack precision

Study	Key results<br>(real-world patients vs RCT-enrolled patients)	Main author conclusions
	respiratory rate lower, and poor renal function was more common	
Golomb et al, 2012 [27]	Self-rated physical activity relative to ‘others the same age’ increased with increasing age in subjects enrolled in an RCT or an observational study	Patients enrolled in an RCT or an observational study may not be representative of the populations they are intended to reflect; selective participation by healthier elderly individuals may potentially influence study outcomes
Hutchinson-Jaffe et al, 2010 [29]	Registry patients with ACS were older and more likely to be female, had significantly more co-morbidities such as hypertension, diabetes, previous MI, HF, and stroke or TIA, and were less likely to be on guideline-	Generalization of ACS clinical trials to real-world patients may be questionable

Study	Key results (real-world patients vs RCT-enrolled patients)	Main author conclusions
	recommended medications or undergo in-hospital procedures	
Melloni et al, 2010 [37]	Only 30% of patients enrolled in RCTs cited by the AHA 2007 Women's Prevention Guidelines were actually female	The proportion of women in RCTs is substantially lower than the proportion of women in the diseased population
Steinberg et al, 2007 [62]	Registry patients with STEMI were older, had more co-morbidities, and were more likely to have a history of cardiac disease; baseline TIMI risk index was similar between RCT and registry patients	While there were baseline differences between registry and RCT patients, outcomes were similar
Uijen et al, 2007[a] [44]	General practice patients with hypertension were	Real-world patients with hypertension differ from RCT patients in

Study	Key results (real-world patients vs RCT-enrolled patients)	Main author conclusions
	older and more likely to female, had a higher risk of cardiovascular disease, and had higher initial SBP and DBP	a number of important characteristics, which may hamper the external validity of the RCTs
Wagner et al, 2011 [65]	Nontrial CABG patients were older and had significantly higher rates of some chronic diseases including CHF, PVD, cerebrovascular disease, COPD, renal disease, and cancer	In many ways, nontrial CABG patients were similar to RCT-enrolled patients
**Mental health**		
Kushner et al, 2009 [57]	Real-world patients with MDD were not significantly different from RCT patients with respect to	While there were some differences between RCT and clinic patients, their clinical significance remains unknown and it is

Study	Key results (real-world patients vs RCT-enrolled patients)	Main author conclusions
	demographics but had a greater severity of depression on some scales and lower scores on a personality scale that assessed preferences for novel experiences	unclear whether RCT results are generalizable to the clinic
Rabinowitz et al, 2003[a] [59]	Real-world patients with psychosis were not significantly different from RCT patients with respect to baseline demographics; RCT patients were more symptomatic, but otherwise the populations were largely similar	RCT and real-world patients were similar for several key variables
Reidel et al, 2005 [60]	Real-world patients with schizophrenia were slightly	RCT patients had a better health status but were generally

Study	Key results (real-world patients vs RCT-enrolled patients)	Main author conclusions
	older, had a longer duration of illness and more previous hospitalizations, and were more likely to suffer from internistic co-morbidities or neurological abnormalities and be discharged on classic antipsychotics	considered representative of the real-world patient population
Surman et al, 2010[a] [42]	Community patients with ADHD had a higher rate of co-morbidities, multiple anxiety disorders, major depression, alcohol or substance dependence, and antisocial personality; overall functioning and socioeconomic status were lower	Findings from clinical trials may have limited external validity for adults with ADHD in the general population, particularly for those with the greatest burden of co-morbid psychopathology

Study	Key results<br>(real-world patients vs RCT-enrolled patients)	Main author conclusions
Zarin et al, 2005[a] [49]	Clinical practice patients with bipolar disorder or schizophrenia were older and more likely to be female or Caucasian	Patients in RCTs do not represent those in clinical practice; most patients in practice are receiving treatments without direct evidence, raising questions about the direct utility of RCTs for guiding treatment decisions
**Oncology**		
Baquet et al, 2009 [52]	Patients enrolled at a higher rate into RCTs included children and adolescents, male patients for nonsex-specific tumour trials, and female patients for sex-specific tumour trials; the highest percentage of patients accrued into RCTs was white females	Rates of RCT patient accrual varied according to demographic and socioeconomic factors

Study	Key results (real-world patients vs RCT-enrolled patients)	Main author conclusions
Elting et al, 2006 [23]	Patients with cancer not participating in RCTs were older and more likely to be female, and were generally in worse health with more chronic co-morbidities and worse performance status	The prognostic differences observed between RCT and real-world patients call into question the generalizability of clinical trial results
Fraser et al, 2011[a] [25]	Real-world patients with breast cancer had a worse disease prognosis, more drug-related toxicity, and lower drug dose intensity	Caution should be used when extrapolating the results of clinical trial data to real-world populations
Jennens et al, 2006 [30]	Real-world patients with NSCLC or colorectal cancer were older	International trials for NSCLC and colorectal cancer are becoming increasingly unsuitable for application to real-world patients due to increasing age discrepancy

Study	Key results (real-world patients vs RCT-enrolled patients)	Main author conclusions
Kalata et al, 2009 [31]	Registry patients with colorectal cancer were significantly older and more likely to be female, and had more severe disease characteristics and worse prognosis	RCT participants were not representative of all cancer patients in a general population
Mengis et al, 2003[a] [38]	Real-world patients with AML were significantly older, had a worse performance status, more infections, more frequent AML-MDS subtypes, and had a lower rate of cytogenetic analysis	RCT-enrolled patients were not representative of the entire AML patient population; data from Phase III studies may not be extrapolated to all patients with AML
van der Linden et al, 2013 [45]	Observational study patients with LA SCCHN were older, more likely to be female, and had poorer	Selective treatment allocation in daily practice limits the generalizability of trial results

Study	Key results (real-world patients vs RCT-enrolled patients)	Main author conclusions
	prognostic factors	
Yennurajalingam et al, 2013 [48]	Outpatients with cancer-related fatigue were significantly older and were more likely to be male and to have higher symptom intensity scores (including pain, depression, anxiety, dyspnoea, and symptom distress)	Differences between RCT patients and outpatients were clinically relevant suggesting that the results of cancer-related fatigue clinical trials cannot be generalized to outpatients being treated in a palliative care clinic
Yessaian et al, 2005 [66]	Age distribution was similar between RCT patients and the general cervical cancer population; a statistically higher proportion of black and Hispanic women were enrolled	Recruitment to cooperative group cervical cancer trials was proportional meaning that these trials provide a unique opportunity to assess outcomes by race in an equal-care setting

[a]Studies that employed Methods A and B; in these studies RCT and real-world populations were compared, the authors then used the eligibility criteria from the RCT of interest to determine how many patients would hypothetically have been eligible or ineligible for that trial. Results presented in this table are for Method A only (see Additional file 4 for Method B results).

ACS: acute coronary syndrome; ADHD: attention deficit hyperactivity disorder; AHA: American Heart Association; AML: acute myeloid leukemia; CABG: coronary artery bypass graft; CHF: congestive heart failure; COPD: chronic obstructive pulmonary disease; DBP: diastolic blood pressure; HF: heart failure; LA SCCHN: locally-advanced squamous cell carcinoma of the head and neck; MDD, major depressive disorder; MDS: myelodysplastic syndrome; MI: myocardial infarction; NSCLC: nonsmall cell lung cancer; NYHA: New York Heart Association; PVD: peripheral vascular disease; RCT: randomized controlled trial; SBP: systolic blood pressure; STEMI: ST-elevation myocardial infarction; TIA: transient ischemic attack; TIMI: Thrombolysis in Myocardial Infarction.
