# Supplementary material for: A literature review on the representativeness of randomized controlled trial samples and implications for the external validity of trial results
Source: Trials. 2015 Nov 3;16:495. doi: 10.1186/s13063-015-1023-4 (PMC4632358; doi:10.1186/s13063-015-1023-4)
Supplement: Additional file 4: — Key results and main author conclusions from studies assessing rates of ineligibility for RCT participation in a real-world patient population (Method B). Detailed description of results and subjective author conclusions from studies included in the review that employed Method B. (PDF 227 kb) [file 13063_2015_1023_MOESM4_ESM.pdf]

Additional file 4. Key results and main author conclusions from studies assessing rates of ineligibility for RCT participation in a real-world patient population (Method B)

Study	% ineligibility[a]	Main reasons for ineligibility	Ineligible vs eligible patients	Main author conclusions
**Cardiology**				
Bahit et al, 2003 [16]	33.6	ND	Older and more likely to be female; higher Killip class IV and rate of previous MI; lower rate of aspirin use, in-hospital catheterization and PCA; longer length of hospitalization	Real-world patients had higher risk characteristics and worse clinical outcomes compared with RCT patients
Bosch et al, 2008 [19]	41.2	Severe hypertension, contraindications to	Older with a higher risk profile	There is a significant discordance between RCTs and clinical practice

Study	% ineligibility[a]	Main reasons for ineligibility	Ineligible vs eligible patients	Main author conclusions
		anticoagulation, prior cerebrovascular accident, and inability to interpret ST-T segment changes		
Collet et al, 2003 [53]	34.0	HF on admission, creatinine clearance ≤30 ml/min, LBBB or pacemaker, and stroke in the previous 2 months	Older and more likely to be female; higher TIMI risk score; less likely to undergo in-hospital coronary angiography or revascularization, or receive glycoprotein IIb/IIIa inhibitors	While a large proportion of patients would be excluded from RCTs for enoxaparin, these patients could still be safely treated in clinical practice
Costantino et al, 2009[b]	66.2	NYHA class I and II, ejection fraction	ND	Patient selection is crucial in RCTs

Study	% ineligibility[a]	Main reasons for ineligibility	Ineligible vs eligible patients	Main author conclusions
[21]		>35%, presence of co-morbidity, age >80 years, and acute events in the previous months		and raises uncertainties about the complete applicability of trial results to clinical practice
Fortin et al, 2006 [55]	1.4–65.5	ND	ND	Patients who meet eligibility criteria for RCTs have a high rate of co-morbid conditions; whether these patients are sampled or excluded should be reported
Koeth et al, 2009 [34]	46.4	Age >75 years, previous stroke, pre-hospital cardiopulmonary resuscitation, impaired renal	Older and more likely to be female, have diabetes or hypertension; less likely to receive early reperfusion	Patients with STEMI included in RCTs may not be representative of patients encountered in everyday

Study	% ineligibility[a]	Main reasons for ineligibility	Ineligible vs eligible patients	Main author conclusions
		function, pre-hospital delay >12 h, STEMI complicated by cardiogenic shock	therapy or aspirin, clopidogrel, statins, ACEIs, and β-blockers within 48 h of admission	practice
Krumholz et al, 2003 [56]	84.5 (NRMI) 90.6 (CCP)	Presentation >6 h after symptom onset and no chest pain or ST-segment elevation on admission, previous stroke, contraindication to thrombolytic therapy	ND	Older patients in the randomized GUSTO trial were similar to patients in clinical practice; the hypothesis that GUSTO enrolled a healthier patient cohort compared with clinical practice is not supported
Lenzen et al, 2005 [35]	61.6	Age, contraindications, and absence of an LVEF measurement	Older and more likely to be female, have co-morbid hypertension, ACS,	Patients enrolled in landmark HF RCTs were a highly selected group

Study	% ineligibility[a]	Main reasons for ineligibility	Ineligible vs eligible patients	Main author conclusions
			and renal insufficiency; less likely to be receiving treatment with ACEIs, β-blockers, or aldosterone antagonists at baseline	and there was a lack of similarity between clinical practice and RCT patients
Masoudi et al, 2003 [36]	67.0	LVEF ≥0.35, left ventricular systolic dysfunction with contraindication, co-morbidity, and age >80 years	ND	There were significant differences between real-world patients and RCT samples; clinicians often have to extrapolate trial findings to populations in which the treatments were not studied
Steg et al, 2007 [40]	33.6	ND	Older with a more frequent history	Caution should be used when

Study	% ineligibility[a]	Main reasons for ineligibility	Ineligible vs eligible patients	Main author conclusions
			of MI, diabetes, TIA or stroke, PAD, and CABG; less likely to undergo coronary angiography and PCI, or to receive aspirin, β-blockers, and reperfusion therapy; had a GRACE risk score in the highest tertile	applying RCT findings to general patients with acute MI
Uijen et al, 2007[b] [44]	53.0	ND	ND	A considerable number of real-world patients with hypertension would not be eligible for typical RCTs, which hampers the external validity of the RCTs

Study	% ineligibility[a]	Main reasons for ineligibility	Ineligible vs eligible patients	Main author conclusions
**Mental health**				
Blanco et al, 2008 [18]	75.8	Duration of the depressive episode <4 weeks and >2 years, other co-morbid Axis I disorder in past 12 months, co-morbid dysthymic disorder, and alcohol or drug abuse disorder in past 12 months	ND	The study findings raise questions about the generalizability of clinical trial results to individuals with MDD in the community
Goedhard et al, 2010 [26]	69.8	Substance abuse, presence of a relevant somatic disorder, abnormal routine laboratory values, and use of more than one	Older and more frequent diagnosis of an Axis II personality disorder	Trial outcomes may not be generalizable to the intended population in clinical practice

Study	% ineligibility[a]	Main reasons for ineligibility	Ineligible vs eligible patients	Main author conclusions
		psychotropic drug		
Hoertel et al, 2013 [28]	58.2 (bipolar) 55.8 (acute mania)	Bipolar: suicide risk, history of substance abuse, and significant medical condition; acute mania: history of substance abuse, suicide risk, and significant medical condition	ND	Traditional RCTs tend to exclude the majority of patients with bipolar disorder limiting the generalizability of their findings
Keitner et al, 2003 [32]	85.5	Diagnosis of bipolar disorder, history of substance abuse, mild depression, medical contraindication, and the use of	ND	The majority of subjects with MDD who apply for RCT participation do not meet eligibility requirements; the results may, therefore, only be

Study	% ineligibility[a]	Main reasons for ineligibility	Ineligible vs eligible patients	Main author conclusions
		prohibited psychotropic medications		applicable to a small subset of patients treated in clinical practice
Khan et al, 2005 [33]	98.2	Requirement for monodrug therapy, male patients only, evidence of substance abuse, obesity, and hepatitis B/HIV	ND	Inclusion and exclusion criteria can restrict the number of eligible patients and affect RCT generalizability
Rabinowitz et al, 2003[b] [59]	33.0	Current antidepressant treatment, substance abuse in the previous month, suicide attempt, and current alcohol abuse	ND	RCT samples and real-world patient populations were largely similar on several key variables
Seemuller et al,	69.0	Low illness severity, co-morbid	Younger, with a trend toward	There were few differences

Study	% ineligibility[a]	Main reasons for ineligibility	Ineligible vs eligible patients	Main author conclusions
2010 [61]		nondepressive, nonsubstance abuse Axis I disorders, significant suicide risk, and substance abuse	younger age at disease onset	between eligible and ineligible patients suggesting that the results from MDD efficacy trials might be more generalizable than previously thought
Storosum et al, 2004 [41]	83.8[c]	No use of contraceptives, use of prior mood stabilizing medication, co-morbid disease, other Axis I diagnosis, co-morbid alcohol or drug use, and suicidal ideation	ND	Few acute manic episodes in a routine mental hospital are eligible for a standard RCT, which may be problematic for the generalizability of trial results to clinical practice
Surman et al,	61.0	ND	Higher rates of lifetime co-	RCT results have limited external

Study	% ineligibility[a]	Main reasons for ineligibility	Ineligible vs eligible patients	Main author conclusions
2010[b] [42]			morbidity, more impaired overall functioning, and lower socioeconomic status	validity for adults with ADHD in the general population
Talamo et al, 2008 [63]	77.6	Substance abuse, suicide attempts and other violent acts within 90 days of index hospital admission, lifetime co-morbid anxiety disorder diagnosis, and involuntary status	Few differences in most demographic and clinical characteristics, except for a slightly higher rate of prior medical illness, shorter lifetime illness, a lower rate of mixed states, and lower initial mania and depression rating scores	Ineligible and eligible patients were similar regarding baseline characteristics suggesting that findings from antimanic treatment RCTs might be relevant to clinical practice
van der Lem et al, 2011[e]	75.5–81.2	Presence of nondepressive,	ND	The influence of eligibility on

Study	% ineligibility[a]	Main reasons for ineligibility	Ineligible vs eligible patients	Main author conclusions
[64]		nonsubstance abuse Axis I disorders, low baseline disease severity, risk of suicide, substance abuse, dysthymic disorder, bipolar or psychotic features, and borderline personality pathology		treatment outcome was small indicating that stringent patient selection may not be the major reason for lack of RCT generalizability
Wisniewski et al, 2009 [47]	77.8	Score of <14 on the 17-item HAM-D and failure to return for first post-baseline visit	Older and less educated; more likely to be black, Hispanic, unemployed, and to have a lower income; longer disease duration; family history of substance abuse;	Patient samples meeting the selection criteria for an RCT are not representative of depressed patients in clinical practice suggesting that RCT outcomes may

Study	% ineligibility[a]	Main reasons for ineligibility	Ineligible vs eligible patients	Main author conclusions
			more suicide attempts and anxious or atypical symptom features	be more optimistic than those obtained in practice
Zarin et al, 2005[b] [49]	55.0 (bipolar) 38.0 (schizophrenia)	Bipolar: substance use, CNS or neuromuscular disorders, and major medical disorder; schizophrenia: childbearing potential and major medical disorder	More co-morbid disease; lower GAF scores; more frequent use of antipsychotic medication or prescribed psychotropic drugs	Patients in RCTs do not represent those in clinical practice, raising questions about the direct utility of RCTs for guiding treatment decisions
Zetin and Hoepner, 2007 [50]	91.4	Insufficient symptom severity, bipolar disorder, co-morbid anxiety disorders, suicidal ideation, and	ND	Effectiveness in patients in clinical practice may be different to outcomes reported in RCTs:

Study	% ineligibility[a]	Main reasons for ineligibility	Ineligible vs eligible patients	Main author conclusions
		depression duration >24 months		uncontrollable factors in RCTs may limit extrapolation of data to real-world practice
Zimmerman et al, 2004 [51]	65.8	Depression rating scale scores below cut-off, anxiety disorder, borderline personality disorder, substance abuse/dependence, dysthymic disorder	ND	RCT patients represent only a minority of patients with MDD treated in the community
**Oncology**				
Clarey et al, 2012 [20]	31.0–76.0	Life expectancy <12 weeks, inadequate performance status,	ND	RCT results may not be generalizable to the majority of

Study	% ineligibility[a]	Main reasons for ineligibility	Ineligible vs eligible patients	Main author conclusions
		abnormal blood results, presence of poor prognostic features, and co-morbidities		patients with advanced NSCLC
Filion et al, 2012 [54]	–[d]	Receptor status not met, pathological criteria not met, other medical condition	ND	The majority of patients with breast cancer who were potentially eligible for inclusion in breast cancer RCTs met the specific eligibility criteria; eligibility criteria were not a large barrier to recruitment in breast cancer RCTs
Fraser et al, 2011[b] [25]	14.9	Age ≥65 years and previous cancer	ND	Caution should be used when

Study	% ineligibility[a]	Main reasons for ineligibility	Ineligible vs eligible patients	Main author conclusions
		diagnosis		extrapolating the results of clinical trial data to real-world populations
Mengis et al, 2003[b] [38]	87.0	Intensive chemotherapy, older than upper age limit, palliative chemotherapy, supportive care, significant co-morbidity, AML subtype, previous history of cancer, investigator decision, MDS, no guaranteed follow-up, and patient refusal	ND	Data from Phase III studies may not be extrapolated to all patients with AML
Mol et al, 2013 [58]	21.5	Poor performance status, serious	Worse performance status; higher	Trial results have external validity

Study	% ineligibility[a]	Main reasons for ineligibility	Ineligible vs eligible patients	Main author conclusions
		co-morbidity, laboratory abnormalities, second malignancy in the past 5 years, no evaluable disease parameter, CNS metastases, and other reasons	levels of alkaline phosphatase; lower rate of primary tumour resection	provided that standard eligibility criteria are observed
Somer et al, 2008 [39]	71.0	Performance status ≥2, CNS metastasis, squamous histology, and anticoagulation/NSAID therapy	ND	Most patients who might have been eligible for standard advanced NSCLC trials were not candidates for ECOG 4599; outcomes from this trial should take into account the eligibility restrictions

Study	% ineligibility[a]	Main reasons for ineligibility	Ineligible vs eligible patients	Main author conclusions
Terschüren et al, 2010 [43]	35.9 (HL) 70.4 (hgNHL)	HL: age >75 or <16 years, reduced compliance, severe co-morbidity, diagnosis of another tumour in the last 5 years, and poor physical condition; hgNHL: age >75 years or <18 years, lactate dehydrogenase value >240 U/l and age >18 years and <60 years, marked impairment of cardiac, pulmonary, hepatic or renal function, NHL of the CNS, and diagnosis of another tumour in the	ND	RCT patients do not represent all patients with hgNHL and HL in the population; trial inclusion criteria caused considerable selection among participants

Study	% ineligibility[a]	Main reasons for ineligibility	Ineligible vs eligible patients	Main author conclusions
		last 5 years		
Vardy et al, 2009 [46]	65.0–72.0	ECOG Performance Status ≥2, co-morbidities, previous cancer history, and symptomatic brain metastasis	ND	The generalizability of RCT results to the general advanced NSCLC population may be limited; results have limited applicability to patients in practice

[a]Percentage of patients not eligible for RCT inclusion following the application of RCT inclusion/exclusion criteria; [b]Studies that employed Methods A and B; in these studies RCT and real-world populations were compared, the authors then used the eligibility criteria from the RCT of interest to determine how many patients would hypothetically have been eligible or ineligible for that trial. Results presented in this table are for Method B only (see Additional file 3 for Method A results); [c]Percentage of manic episodes not number of patients that would have been ineligible; [d]Inclusion/exclusion criteria were categorized in order to identify criteria that might impede RCT recruitment; if any individual category was not met by >10% of patients with breast cancer from a retrospective cohort, then the criterion was

considered a barrier to recruitment; [e]75.5% based on application of stringent criteria using the Mittman regression equation to calculated HAM-D; 81.2% based on application of stringent criteria using the Hawley or Zimmerman regression equation to calculate HAM-D.

ACEIs: angiotensin-converting enzyme inhibitors; ACS: acute coronary syndrome; ADHD: attention deficit hyperactivity disorder; AML: acute myeloid leukemia; CABG: coronary artery bypass graft; CCP: Cooperative Cardiovascular Project; CNS: central nervous system; ECOG: Eastern Cooperative Group; GAF: Global Assessment of Functioning; GRACE: Global Registry of Acute Coronary Events; GUSTO: Global Utilization of Streptokinase and t-PA for Occluded Coronary Arteries; HAM-D: Hamilton Depression Rating Scale; HF: heart failure; HL: Hodgkin's lymphoma; hgNHL: high-grade nonHodgkin's lymphoma; LBBB: left-branch bundle block; LVEF: left ventricular ejection fraction: MDD: major depressive disorder; MDS: myelodysplastic syndrome; MI: myocardial infarction; ND: not determined; NRMI: National Register of Myocardial Infarction; NSAID: nonsteroidal anti-inflammatory drug; NSCLC: nonsmall cell lung cancer; NYHA: New York Heart Association; PAD: peripheral arterial disease; PCA: percutaneous coronary angioplasty; PCI: percutaneous coronary intervention; RCT: randomized controlled trial; STEMI: ST-elevation myocardial infarction; TIA: transient ischemic attack; TIMI: Thrombolysis in Myocardial Infarction.
